# Research on the pathogenesis of Alzheimer's disease based on thalamocortical computational model

**DOI:** 10.3389/fneur.2024.1353305

**Published:** 2024-04-24

**Authors:** Yijin Gang, Tao Li, Xianjing Xu, Qi Zheng, Weiping Wang

**Affiliations:** ^1^School of Human Settlements and Civil Engineering, Xi'an Jiaotong University, Xi'an, China; ^2^Department of Cardiovascular Medicine, Henan Province People's Hospital, Zhengzhou, Henan, China; ^3^School of Computer and Communication Engineering, University of Science and Technology Beijing, Beijing, China; ^4^Shunde Innovation School, University of Science and Technology Beijing, Foshan, China

**Keywords:** Aβ amyloid protein, Alzheimer's disease, inhibitory neuron population, alpha rhythm, EEG

## Abstract

Alpha rhythm slowing is an important electroencephalogram(EEG) feature associated with (AD). This study aims to understand the correlation between alpha band deceleration and molecular changes from the perspective of neural computing. Considering the effect of Aβ amyloid deposition on the inhibitory changes in the thalamic, a thalamic cortical model coupled with Aβ amyloid is established. The results show that Aβ amyloid deposition may induce neurotoxicity in thalamic reticular nucleus neurons, which results in inhibitory changes in the thalamus and slows the alpha rhythm of EEG output from the thalamus. In order to understand the pathogenesis more intuitively, some numerical simulations are provided to illustrate the obtained theories. This research is helpful to understand the pathogenesis of AD, so as to provide theoretical basis for the intervention and control of the disease.

## 1 Introduction

As a common degenerative neurological disease, Alzheimer's disease (AD) seriously threatens human health (1). Statistics show that about 50%–60% of dementia patients are related to AD. It is caused by many complex factors, and its phenomenon is mainly manifested in the decline of cognitive ability (2). Therefore, the early diagnosis and clinical intervention of AD have attracted great attention of the society. At present, the neuronal mechanisms related to the pathogenesis of AD remain unclear. Modeling can help to understand the temporal and spatial characteristics of various neurological diseases, so as to predict dynamic trends(3). To gain a clearer understanding of the biomolecular and neuron-related mechanisms of AD, many people are increasingly turning their attention to computational models related to neurological or psychiatric disorders(4).

The slowing of alpha band is a common biomarker of AD (5, 6). Therefore, many computational models based on neural field are proposed to simulate EEG signals (7, 8). Bhattacharya found that inhibitory neuronal population plays an important role in normal brain activity by using the computational model of thalamic cortical neuronal population loop related to AD (9). Recently, Stefanovski incorporated the effect of Aβ amyloid on neuronal population dynamics into the Jason Ritter network model through neural computing method,which linked Aβ amyloid related synaptic disinhibition to specific alpha rhythm slowing in EEG. The neurobiological processes more directly related to the pathogenesis can be identified by neural computing model (10). The inhibitory effect of thalamic reticular nucleus neuronal population (TRN) on thalamic relay nucleus neuronal population (TCR) and the deinhibitory effect of Aβ amyloid protein on inhibitory neuronal population provide ideas for the realization of inhibitory damage in this study.

The aggregated Aβ amyloid and its related intermediates cause damage to the inhibitory synaptic plasticity in the neural circuit (11–13). This suggests that the impairment of inhibitory neuronal populations in the brain network leads to Alzheimer's disease (14). In Stefanovski's study, only the disinhibition of Aβ protein deposition on the inhibitory interneuron population in the cortical module was considered, resulting in the overexcitation of excitatory pyramidal neuron population. However, some studies have shown that thalamic relay nucleus neurons have excitatory relay effects on pyramidal neurons (15). Based on the inhibitory effect of the inhibitory thalamic reticular nucleus on the thalamic relay nucleus (16), we decided to integrate the mapping function of Aβ amyloid protein in Stefanovski's study into the thalamic reticular nucleus of the thalamic module to achieve disinhibition.

According to the above effect of Aβ amyloid on inhibitory neurons, the ratio of excitability to inhibition time constant of thalamic cortical circuit model can also simulate the effect of Aβ amyloid on inhibitory neurons. All these make it possible to simulate the disinhibition affected by Aβ amyloid in the thalamic cortical circuit. Meanwhile,the model can well simulate EEG of brain. The specific steps of this study are as follows. First, we introduced a thalamic cortical model influenced by Aβ amyloid and obtained preliminary simulation results. Then the effects of Aβ amyloid and synaptic connection parameters on the power spectrum from the model output is discussed. Finally, this study is summarized.

## 2 Methods

According to the introduction, our goal is to map the effect of Aβ amyloid deposition to the thalamic cortical neural calculation model proposed by Bhattacharya (9). The model consists of TCR representing excitatory neuron population and TRN representing inhibitory neuron population. TRN neuron population has inhibitory projection to TCR neuron population. On the contrary, TCR neuron population has excitatory feedback to TRN neuron population and they communicate through synapses (17, 18) Based on Stefanovski's recent research (10), we tried to map the effect of Aβ amyloid deposition on the inhibition time constant to the inhibitory neuron population in the thalamic cortical neural calculation model. The thalamic cortical equation and the mapping function equation from Aβ amyloid to inhibition time constant are as follows.


(1) $$\begin{array}{l} \;{ẋ}_{ret1}=x_{ret2}\\ {ẋ}_{ret2}=\frac{H_{e}}{\tau_{e}}P\left(t\right)-\frac{2}{\tau_{e}}x_{ret2}-\frac{1}{\tau_{e}^{2}}x_{ret1} \end{array}$$

(2) $$\begin{array}{l} \;{ẋ}_{tcr1}=x_{tcr2}\\ {ẋ}_{tcr2}=\frac{H_{e}}{\tau_{e}}S\left(C_{3}x_{ret1}-C_{2}x_{trn1}\right)-\frac{2}{\tau_{e}}x_{tcr2}-\frac{1}{\tau_{e}^{2}}x_{tcr1} \end{array}$$

(3) $$\begin{array}{l} \;{ẋ}_{trn1}=x_{trn2}\\ {ẋ}_{trn2}=\frac{H_{i}}{\tau_{i}\left(\beta_{a}\right)}S\left(C_{1}x_{trn1}\right)-\frac{2}{\tau_{i}\left(\beta_{a}\right)}x_{trn2}-\frac{1}{\tau_{i}{\left(\beta_{a}\right)}^{2}}x_{trn1} \end{array}$$

(4) $$\begin{array}{l} S\left(Vcell\right)=\frac{2e_{0}}{1+e^{-\nu \left(Vcell-s_{0}\right)}} \end{array}$$

In the model equation, *x*_*ret*_,*x*_*tcr*_ and *x*_*trn*_ are the state variables of retina, thalamic relay nucleus and thalamic reticular nucleus respectively. *H*_*e*_,*H*_*i*_ represent the synaptic strength of excitatory and inhibitory postsynaptic potential. τ_*e*_ is the excitability time parameter. τ_*i*_(β_*a*_) is the inhibitory time parameter affected by Aβ amyloid deposition. Each connectivity parameter *C*_*i*_: i={1,2,3} represents the connectivity parameter generated by presynaptic neuron group. *C*_1_ represents the excitatory connection from relay nucleus to reticular nucleus *C*_*nte*_, *C*_2_ represents the inhibitory connection from reticular nucleus to relay nucleus *C*_*tni*_, and *C*_3_ represents the external excitatory input of relay nucleus *C*_*tre*_. The exogenous input of thalamus module is represented by P(t). With mean μ_*r*_and variance ϕ_*r*_, Gaussian white noise is used to simulate P(t). The average potential of the post synaptic is converted into action potential pulse density by a sigmoid function *S*(·). the maximum discharge rate is 2*e*_0_ and *s*_0_ the discharge threshold. The slope of *S*(·) is ν. See Table 1 for specific parameter values.

**Table 1:** The relevant parameters are defined in Equations (1)–(5), and all parameters are derived from the relevant work of Bhattacharya et al. (9).

**Parameter**	**Value**
*H*_*e*_(*mv*)	3.25
*H*_*i*_(*ms*)	22
$\tau_{e}\left(mV^{-1}\right)$	10
ν(*s*^−1^)	0.56
*s*_0_	6
*e*_0_	25
*C*_1_(%)	35
*C*_2_(%)	15
*C*_3_(%)	7
μ_*r*_(*sps*)	0 ≤ μ_*r*_ ≤ 100
$\phi_{r}\left(sps^{2}\right)$	1

The output is the post-synaptic potential of the TRN in the model, in which its equation is defined as:


(5) $$\begin{array}{l} V_{tcr}=C_{3}x_{ret1}-C_{2}x_{trn1} \end{array}$$

The mapping function of amyloid Aβ to inhibitory time constant:


(6) $$\begin{array}{l} \;\tau_{i}\left(\beta_{a}\right)=S_{1}^{-1}\left(\beta_{a}\right)\\ r_{\beta_{a}}=2\ln\left(S_{max}\cdot 1s-1\right)/\left(\beta_{a,off}-\beta_{a,max}\right)\\ \;\beta_{0}=\left(\beta_{a,off}+\beta_{a,max}\right)/2 \end{array}$$

(7) $$\begin{array}{l} S_{1}\left(\lambda \right)=\frac{S_{max}-S_{min}}{1+exp^{\left(r_{\lambda }\left(\lambda_{0}-\lambda \right)\right)}}:0<S_{min}<S_{max} \end{array}$$

In the mapping function of Aβ amyloid to inhibitory time constant, β_*a*_ is the current local Aβ amyloid load measured by PET. β_*a, off*_ is the threshold cut-off value of Aβ amyloid, which is used to distinguish normal and pathological Abeta load. β_*a, max*_ is the maximum possible load value of β_*a*_ amyloid detected by PET in the population. Sigmoid function *S*_1_(·) is a continuously differentiable decreasing conversion function, which is used to convert the load value of β_*a*_ detected by PET into inhibitory time constant τ_*i*_(β_*a*_) and realize the mapping from Aβ amyloid to inhibitory time constant. *r*_β_*a*__ and β_0_ are the slope and midpoint of sigmoid function respectively. Aβ amyloid loading affects the inhibitory time constant, which follows the sigmoid function curve. The variation changes from *S*_*min*_ to *S*_*max*_. Therefore, the inhibitory dendritic time constant τ_*i*_(β_*a*_) varies from 1/*S*_*max*_ to 1/*S*_*min*_. See Table 2 for specific parameter values.

**Table 2:** The specific values of the parameters defined in Equations (6), (7), and the values of all parameters are derived from stefanovski's relevant work (10).

**Parameter**	**Value**
β_*a, max*_	2.65
β_*a, min*_	1.4
τ_*i*_(β_*a*_)(*ms*)	14.29 ≤ τ_*i*_(β_*a*_) ≤ 50
*S*_*max*_	0.07
*S*_*min*_	0.02

The related equations defined in the model are calculated in MATLAB by fourth-order/fifth-order Runge-Kutta method. The total simulation time is 30s. The parameter values of each population were repeated for 20 times to generate the membrane potential of thalamic relay nucleus neurons and the average value was taken to ensure the accuracy of statistics. It is necessary to better extract the features of EEG signal by computer technology analysis and other auxiliary techniques. The power spectrum analysis of EEG signals is an useful means to study Alzheimer's disease (19). To get the power spectral density of alpha (8–13Hz) frequency band of thalamic output, we analyzed the membrane potential of thalamic relay nucleus neuron population: (1) The membrane potential is sampled and bandpass filtered by a butterworth filter. (2) Welch period graph method is used to calculate the power spectrum (20).

## 3 Results

### 3.1 Regulate Aβ amyloid standardized uptake value ratio (SUVR) β_*a*_

Stefanovski showed that the standardized uptake value ratio (SUVR) of Aβ amyloid affects the inhibitory time constant of inhibitory neuron population. The SUVP is expressed as parameter β_*a*_ in the above formula (10). In this study, the inhibitory time constant is affected by changing the deposition amount of Aβ amyloid in the thalamic cortical model. The alpha rhythm of the model output signal is observed.

When β_*a*_ is lower than the clinical critical value of 1.4, the τ_*i*_(β_*a*_) is not affected. the corresponding τ_*i*_(β_*a*_) is 14.29ms(21). Therefore, we default that the τ_*i*_(β_*a*_) = 14.29ms is not affected by Aβ amyloid. This study believes that there is no abnormal pathology in such brain regions. When β_*a*_ is between 1.4 and 1.95, the corresponding τ_*i*_(β_*a*_) is between 14.29ms and 20ms. In this state, these regions correspond to moderate plogical state of Aβ amyloid in the brain region. When β_*a*_ is between 1.96 and 2.15, the corresponding τ_*i*_(β_*a*_) is between 20ms and 28ms. At this time, these areas are moderate to severe Aβ amyloid pathological state (21). When there is a serious pathological state of Aβ amyloid in the brain region,the time constant is greater than 28ms. β_*a*_ will be greater than 2.15.

**Figure 1:**
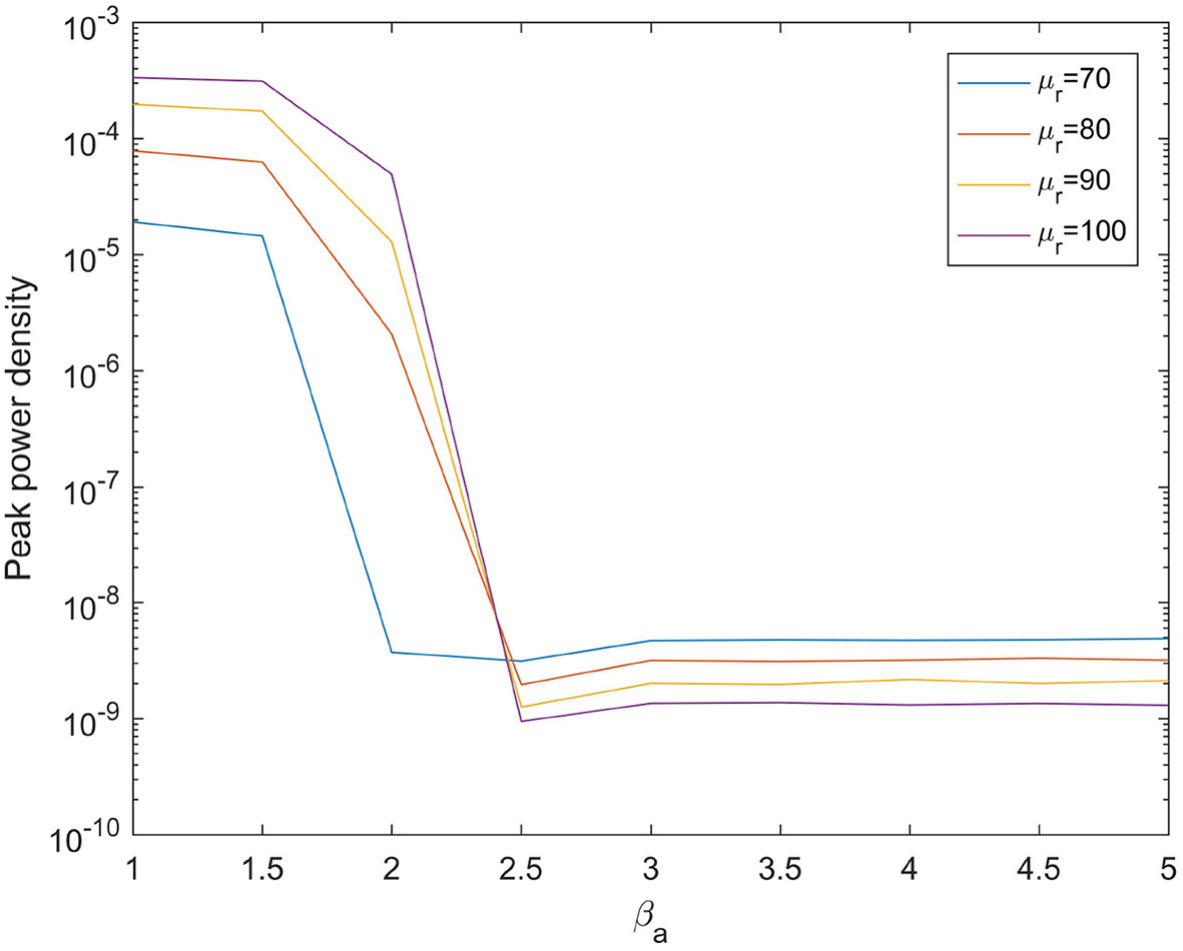
When μ_*r*_={70,80,90,100}, the variation trend of the peak power of the signal output from the model with the change of β_*a*_.

When the mean value μ_*r*_ of input noise is arbitrarily selected, the power spectral density analysis is performed based on the signal output from the above model. Figure 1 demonstrates the influences of β_*a*_ on the peak power of the signal output from the model. The change range of β_*a*_ is 1.4-5.0, which corresponds to the state of normal to seriously abnormal pathological brain. The experimental results show that when the noise mean value μ_*r*_ is randomly selected, the peak power output from the calculation model decreases with the increase of β_*a*_, and finally tends to be relatively stable.

**Figure 2:**
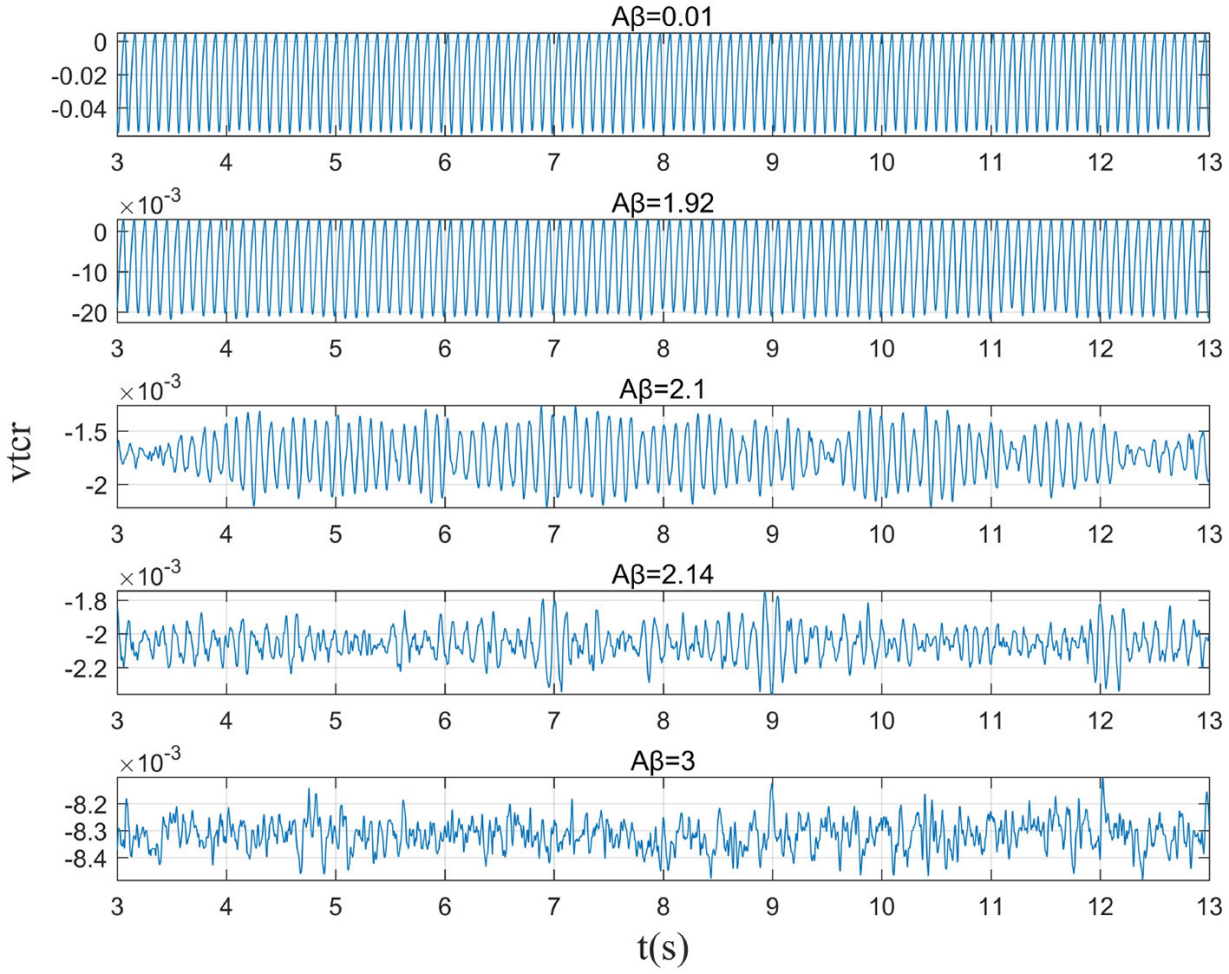
When μ_*r*_=86, the time series of the signal output from the model when β_*a*_={0.001,1.92,2.10,2.14,3.00}.

**Figure 3:**
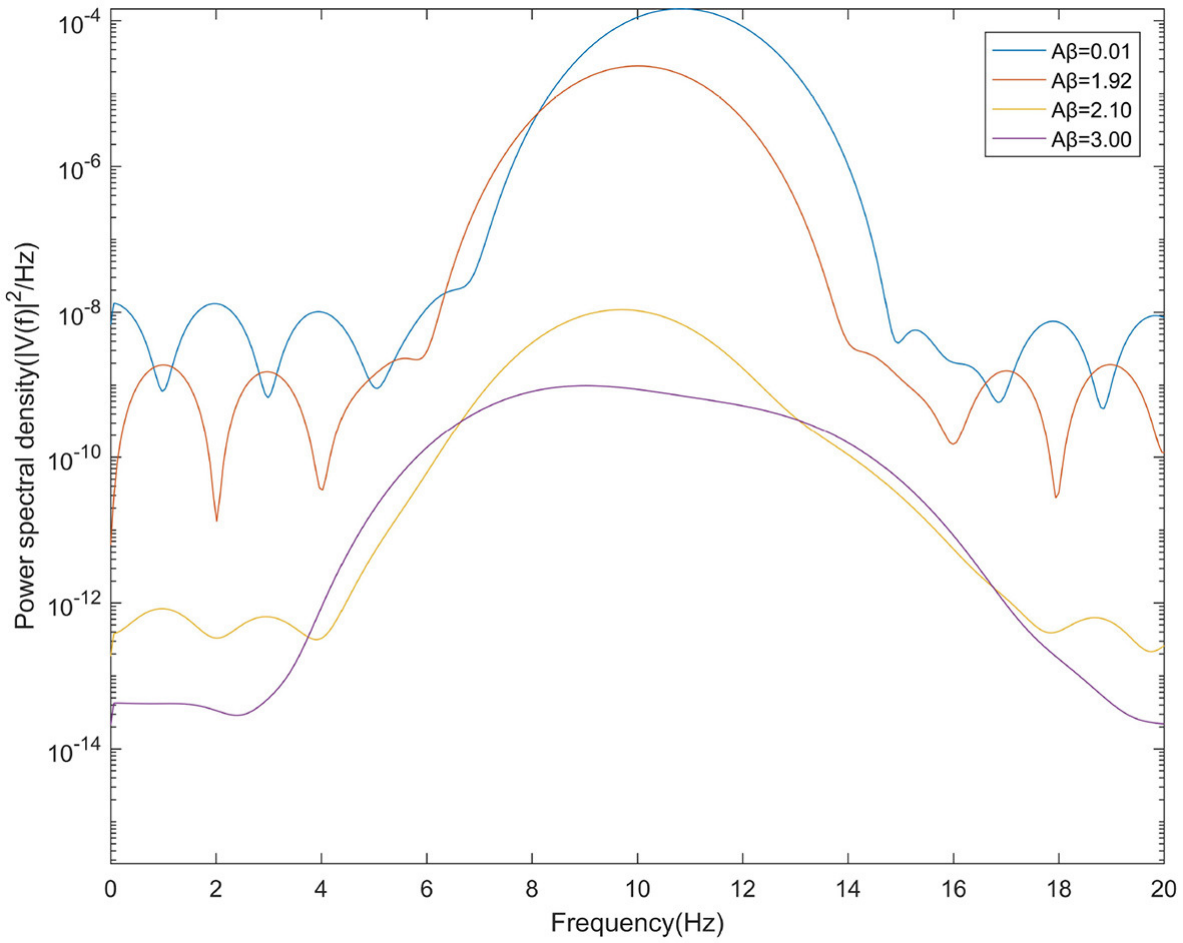
When μ_*r*_=86, power spectral density of the signal output from the model when β_*a*_={0.001,1.92,2.10,3.00}.

**Figure 4:**
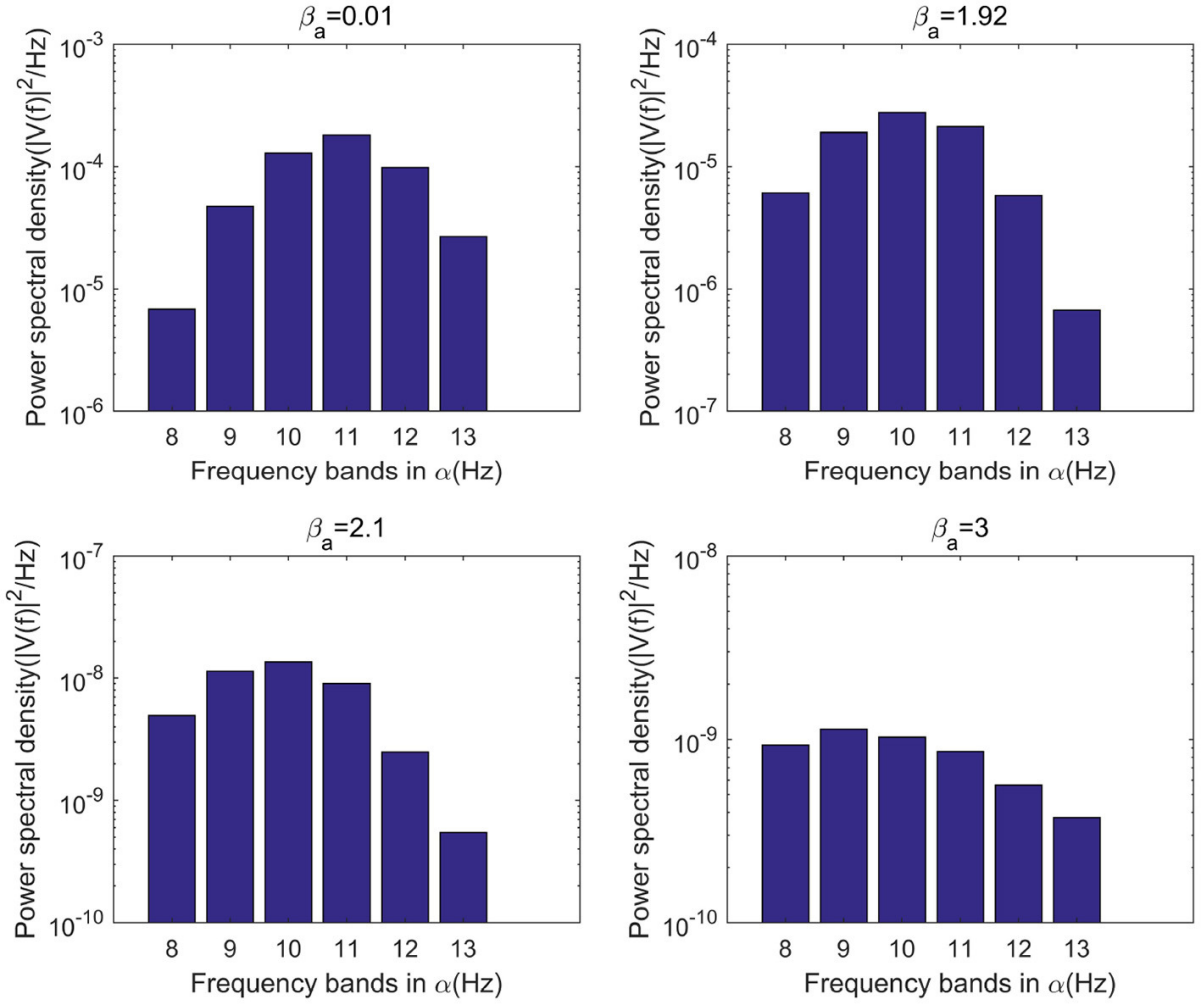
When μ_*r*_=86, bar graph of power of each frequency band within α frequency band of the signal output from the model when β_*a*_={0.01,1.92,2.10,3.00}.

This study further analyzes the effect of Aβ amyloid on thalamic cortical model. Figure 2 shows the time series of signals output under the influence of Aβ amyloid. Figure 3 demonstrates the power spectral of the model output signal corresponding to some values. It can be seen from the figure that with the increase of Aβ amyloid deposition in thalamus, the corresponding peak power and dominant frequency within alpha band decrease. It can also be seen from the figure that this change is very small in moderate diseases, but very significant in the pathology of severe diseases. As can be seen from Figure 4, with the increase of parameter related to Aβ amyloid in the model, the power of each frequency band within alpha band decreases. The result shows that when the parameter related to Aβ amyloid increased in the thalamic cortical model, the alpha rhythm output from the model slowed down.

### 3.2 Regulate the synaptic connection parameter *C*_1_ from TCR to TRN

In Bhattacharya's study, the increase of excitability parameter C1 indicates that the synaptic activity of TCR neurons to TRN neurons is increased (22) In this section, the value of excitability parameter *C*_1_ was changed between 0 and 100 in the state of moderate to severe disease. Then the effect on the alpha band was observed through power spectrum analysis.

**Figure 5:**
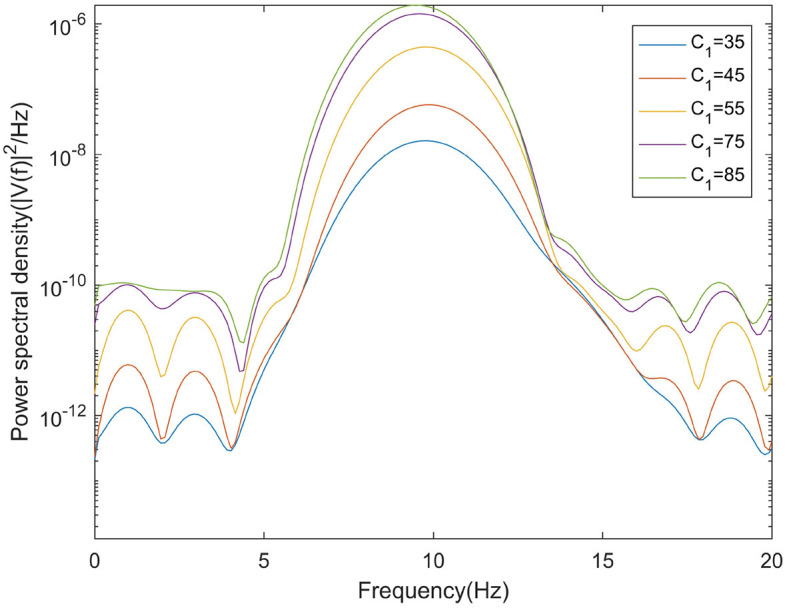
When μ_*r*_ = 86, β_*a*_ = {2.10}, the power spectral of the signal output from the model varying with *C*_1_.

**Figure 6:**
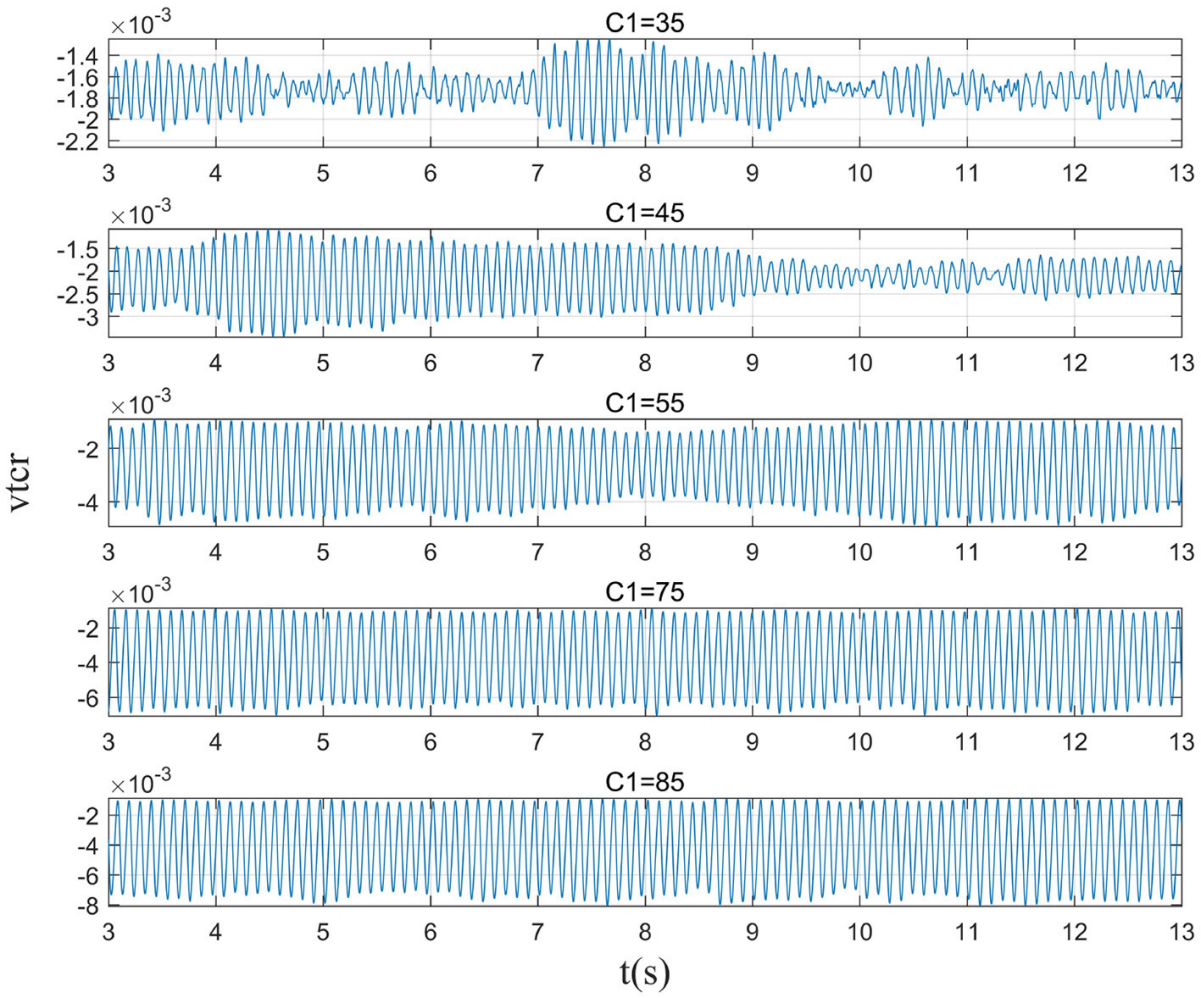
When μ_*r*_ = 86,β_*a*_ = {2.10}, the time series of the signal output from the model varying with *C*_1_.

In moderate to severe illness, *C*_1_ value is increased on the basis of excitability parameter *C*_1_ = 35. Alpha band power is analyzed through power spectrum analysis. It can be observed from Figure 5 that with the increase of *C*_1_ value, the corresponding alpha band peak power in the power spectrum increases significantly. The time series corresponding to the EEG signal output from the model is shown in Figure 6. The results showed that in moderate to severe disease, the increase of the excitatory synaptic activity in the afferent pathway from TCR neuron population to TRN neuron population can lead to peak power within alpha band increase significantly. This may indicate that when Aβ amyloid deposition destroys the inhibitory of TRN neuron population, excitatory synaptic activity of TRN neuron population afferent pathway can enhance the inhibitory of TRN neuron population.

### 3.3 Regulate Gaussian white noise mean μ_*r*_

The mean μ_*r*_ of Gaussian white noise is a key parameter in the sensory pathway of thalamic cortical model. Higher input value means that the activity of sensory pathway increases, which may indicate the recovery of sensory information related to eye opening. Therefore, this section explores the effect of μ_*r*_ on the peak power of the alpha band by adjusting the mean μ_*r*_ of Gaussian white noise in the moderately to severely ill states.

**Figure 7:**
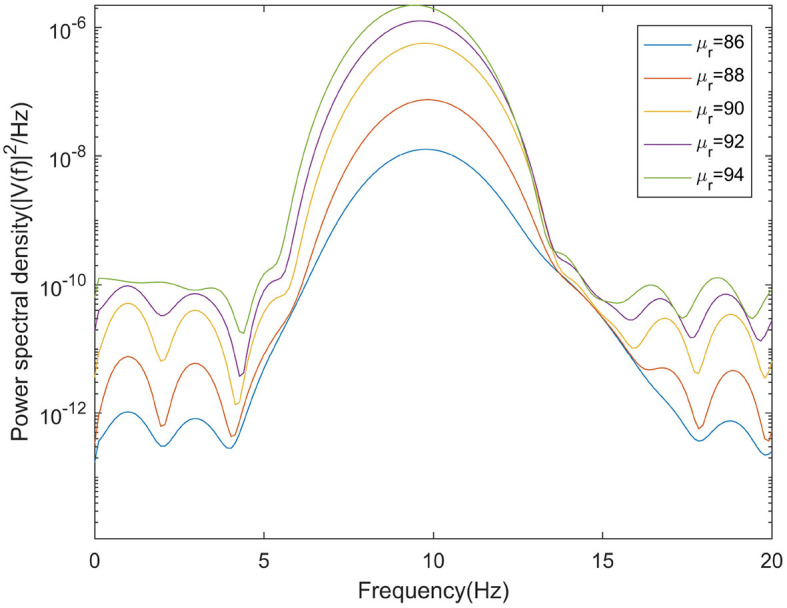
When β_*a*_ = {2.10}, the power spectral of the signal output from the model varying with μ_*r*_.

**Figure 8:**
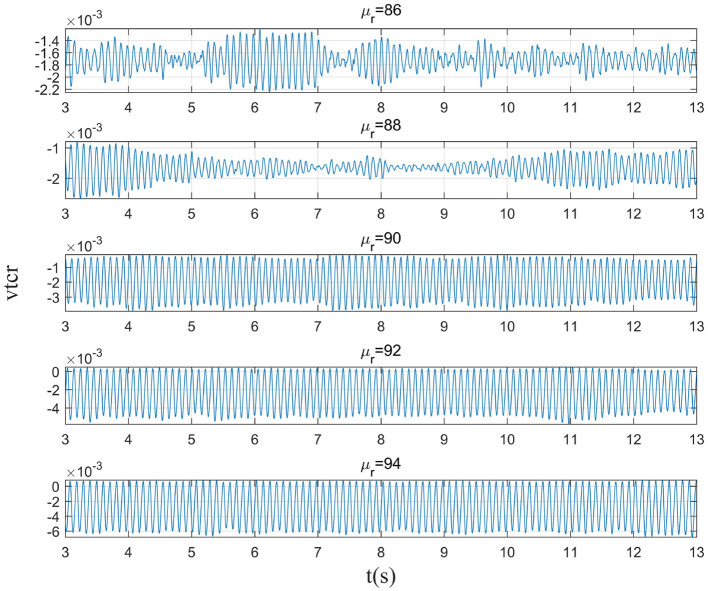
When β_*a*_ = {2.10}, the time series of the signal output from the model varying with μ_*r*_.

This section mainly simulates the enhancement of external stimuli by increasing the mean value of Gaussian white noise based on μ_*r*_ = 86. The corresponding changes of EEG signal and peak power spectrum is observed under different mean values μ_*r*_. With the increase of μ_*r*_, the growth of the peak power in the alpha band can be seen from Figure 7. The result shows that in moderate to severe diseases, the increase of external stimulation of TCR neurons could significantly increase the peak power spectrum of alpha band. It can be observed in Figure 8 that the time series corresponding to the mean values of different input noise.

## 4 Conclusions

This study mainly consideres the neurotoxicity of Aβ amyloid deposition to thalamic reticular nucleus, giving rise to damage on TRN inhibitory function. This study firstly consideres the effect of Aβ amyloid deposition on the inhibition time constant of thalamic reticular nucleus, and integrated it into the thalamic cortical circuit model. By increasing the parameter β_*a*_ related to the content of Aβ amyloid deposition, the disinhibitory neuropathology of Alzheimer's disease caused by Aβ amyloid deposition is simulated. Then the power spectrum of the signal output from the thalamus is analyzed to explore the potential neural mechanism related to the abnormal changes of alpha band.

The results show that thalamic reticular nucleus neuron population play an important role in maintaining normal thalamic concussion. Aβ amyloid deposition will produce neurotoxicity to thalamic reticular nucleus neuron population, resulting in inhibitory damage. The peak power spectral as well as dominant frequency in the α band are reduced.

In addition, in moderate to severe disease, the increase of excitatory synaptic activity in the afferent pathway from TCR to TRN promotes the increase of the peak power in the α band. This may indicate that increasing the excitatory input in the afferent pathway of TRN neuron population can enhance the inhibitory function of TRN neuron population. By increasing the mean value μ_*r*_ of excitatory input in the sensory pathway, the power spectral in the α band also increased significantly.

## 5 Despite the notable findings, the study has limitations

This study investigates the impact of Aβ amyloid deposition on the inhibition time constant of the thalamic reticular nucleus (TRN) and utilizes power spectrum analysis to explore the potential neural mechanisms related to abnormal changes in the alpha band, providing a valuable perspective for understanding the neuropathology of Alzheimer's disease (AD). Although this research offers important insights into the role of Aβ amyloid deposition in AD, the pathology of AD involves a broader range of factors, including tau protein aggregation, neuroinflammation, and oxidative stress. This study focuses on the effects of Aβ amyloid and does not account for these factors that could offer additional insights into the causes of AD. In future research, a more comprehensive model will be developed that includes key factors beyond Aβ amyloid deposition to more fully simulate the complex pathology of AD. The development of such an integrated model will aid in a deeper understanding of the multifactorial pathological mechanisms of AD and in exploring new therapeutic strategies.

## Data availability statement

The original contributions presented in the study are included in the article/supplementary material, further inquiries can be directed to the corresponding author.

## Author contributions

YG: Investigation, Writing—review & editing. TL: Writing—review & editing. XX: Writing—review & editing. QZ: Writing—original draft, Writing—review & editing. WW: Writing—review & editing.
